# Characterization of *Bacillus thuringiensis* isolated from soils in the Jazan region of Saudi Arabia, and their efficacy against *Spodoptera littoralis* and *Aedes aegypti* larvae

**DOI:** 10.1016/j.sjbs.2023.103721

**Published:** 2023-06-28

**Authors:** Usama M. Abu El-Ghiet, Salah A. Moustafa, Mousa M. Ayashi, Mohamed A. El-Sakhawy, Abeer Ali El-Sherbiny Ateya, Hisham Ali Waggiallah

**Affiliations:** a Biology Department, Faculty of Science, Jazan University, Saudi Arabia; b Agriculture Genetic Engineering Research Institute (AGERI), Agricultural Research Center (ARC), Giza 12619, Egypt; c Department of Medical Laboratory Sciences, College of Applied Medical Sciences, Prince Sattam bin Abdulaziz University, Al-Kharj 11942, Saudi Arabia; d Department of Medicinal and Aromatic Plants, Desert Research Center, Cairo, Egypt

**Keywords:** *Aedes aegypti*, *Bacillus thuringiensis*, Characterization, Bioinsecticides, *Spodoptera littoralis*

## Abstract

Pest control in Saudi Arabia depends on applying chemical insecticides, which have many undesirable considerations and impacts on the environment. Therefore, the aim of this study was to isolate *Bacillus thuringiensis* from different rhizosphere soil samples in the Jazan region for the biological control of *Spodoptera littoralis* and *Aedes aegypti* larvae. The samples were collected from the rhizosphere of different plants located in eight agricultural areas in Jazan, Saudi Arabia. Out of 100 bacterial isolates, four bacterial isolates belonging to *Bacillus* species were selected namely JZ1, JZ2, JZ3, and JZ4, and identified using classical bacteriological and molecular identification using 16S *rRNA*. JZ1 and JZ2 isolates were identified as *Bacillus thuringiensis*. SDS-PAGE analysis and the detection of the *Cry1* gene were used to describe the two isolates JZ1 and JZ2 in comparison to *Bacillus thuringiensis* reference strain *Kurstaki HD1* (BTSK) were revealed that slightly different from each other due to the place of their isolation and namely Khlab JZ1 and Ayash JZ2. The EC_50_ of JZ1 and JZ2 isolates, BTSK*,* and the commercial biopesticide DiPEL 6.4 DF against the second-instar larvae of *Aedes aegypti* were 207, 932, 400, and 500 ppm respectively, while EC_50_ against first-instar larvae of *Spodoptera littoralis* were 193.93, 589.7, 265.108, and 342.9, ppm respectively. Isolate JZ1 recorded the highest mortality while JZ2 isolate gave the lowest mortality. It can be concluded that the local isolate of JZ1 and JZ2 can be developed for bio formulations to be used in *Spodoptera littoralis* and *Aedes aegypti* biological control programs.

## 1 Introduction

By the year 2050, it is anticipated that plant protection in modern agriculture will require intensive pesticide application to increase agricultural crop production by 70% in order to feed the growing population (Diouf, 2009). Insect pest resistance emerged as a result of the ongoing use of chemical pesticides, which had numerous negative effects on the environment. Human and other non-target organism safety in biological control. Its natural presence in the environment, lower price, and greater environmental friendliness are just a few of its benefits (Azize et al., 2021), Utilizing pesticides successfully controls harmful organisms without toxicity or negative health effects, simplifying cultivation systems and crop protection strategies, and enabling an increase in yields. Biopesticides are the most significant alternatives to the use of chemical plant protection products (Šunjka and Mechora, 2022). The use of plant protection products that are biochemical, macrobiological, microbiological, and in order to control pests, weeds, and disease-causing organisms is referred to as biopesticides. Furthermore, advantageous microorganisms create vitamins, enzymes, and plant hormones that can strengthen plants' immune systems. These substances are widely used, making up about 30 percent of the production and sales of biopesticides (Grahovac, 2014, Kumar et al., 2021). Certain types of specific species or mixtures of various bacteria, fungi, viruses, or protozoa are present in some microbiological pesticides (Šunjka and Mechora, 2022). Biopesticide based on *Bacillus thuringiensis* (BT) is a type of bacteria that produces proteins toxic to certain insects. These proteins, called BT toxins, can be used to control insect pests in agriculture. BT biopesticides are considered to be environmentally friendly because they target specific pests and do not harm beneficial insects or other organisms. They are also less toxic to humans and animals than synthetic pesticides. BT biopesticides can be applied in several ways, including sprays, dust, and genetically modified plants that express BT toxins (Carozzi et al., 1991, Chilcott and Wigley, 1993, Li et al., 2022). These BT-toxins are specific to certain groups of insects, such as Lepidoptera (moths and butterflies), Coleoptera (beetles), and mosquitoes that transmit diseases like dengue and malaria to humans (Bravo et al., 2011, Kumari et al., 2022). BT is a naturally occurring soil-dwelling bacterium that produces a variety of proteins called *Cry* toxins (Cyt toxins) that are toxic to certain insects. These toxins are used to control pests in agriculture, as they specifically target certain insects and do not harm beneficial insects or mammals (Zafar et al., 2022). Insecticidal proteins known as *Cry* or Cyt toxins are produced by BT during its sporulation phase of growth. These toxins make BT the best microbial insecticide until now (Bel et al., 2020). The mode of action of BT toxin started with *Cry*-protoxins should be converted into active toxins by proteases then bind to specific receptors in the gut of the insect, forming a pore that disrupts the membrane insect gut integrity and ultimately leading to the insect's death. The specificity of the toxin for certain insects is due to the fact that it only binds to receptors found in the gut of those insects, which makes these toxins selectively toxic to the host (Liao et al., 2022, Best et al., 2022). The development of BT crops that express the *Cry* gene resulted in crops that resist insect attack, including borers that were challenging to control with topical BT formulations, and this led to the commercial release of BT crops, which is how BT became successful as a bioinsecticide (Sanahuja et al., 2011). Therefore, still search for new and native strains of BT to produce native and effective toxins is the main concern of many scientists as well as companies working in this field (Liang et al., 2022). The resources of BT strains were dug up in many countries, and thousands of BT strains were isolated. Finding native species of toxic BT strains that can be used for pest management would be interesting. The natural occurrence of BT species with insecticidal activity in the Saudi Arabian environment is not well understood. Finding native species of toxic BT strains that can be used for pest management would be interesting. Finding BT isolates from various soils in Jazan, Saudi Arabia, characterizing these strains using molecular techniques, and determining their larvicidal activity against *Spodoptera littoralis* and *Aedes aegypti* larvae were the objectives of this study.

## 2 Materials and methods

### 2.1 Collection and processing of soil samples

**Fig. 1:**
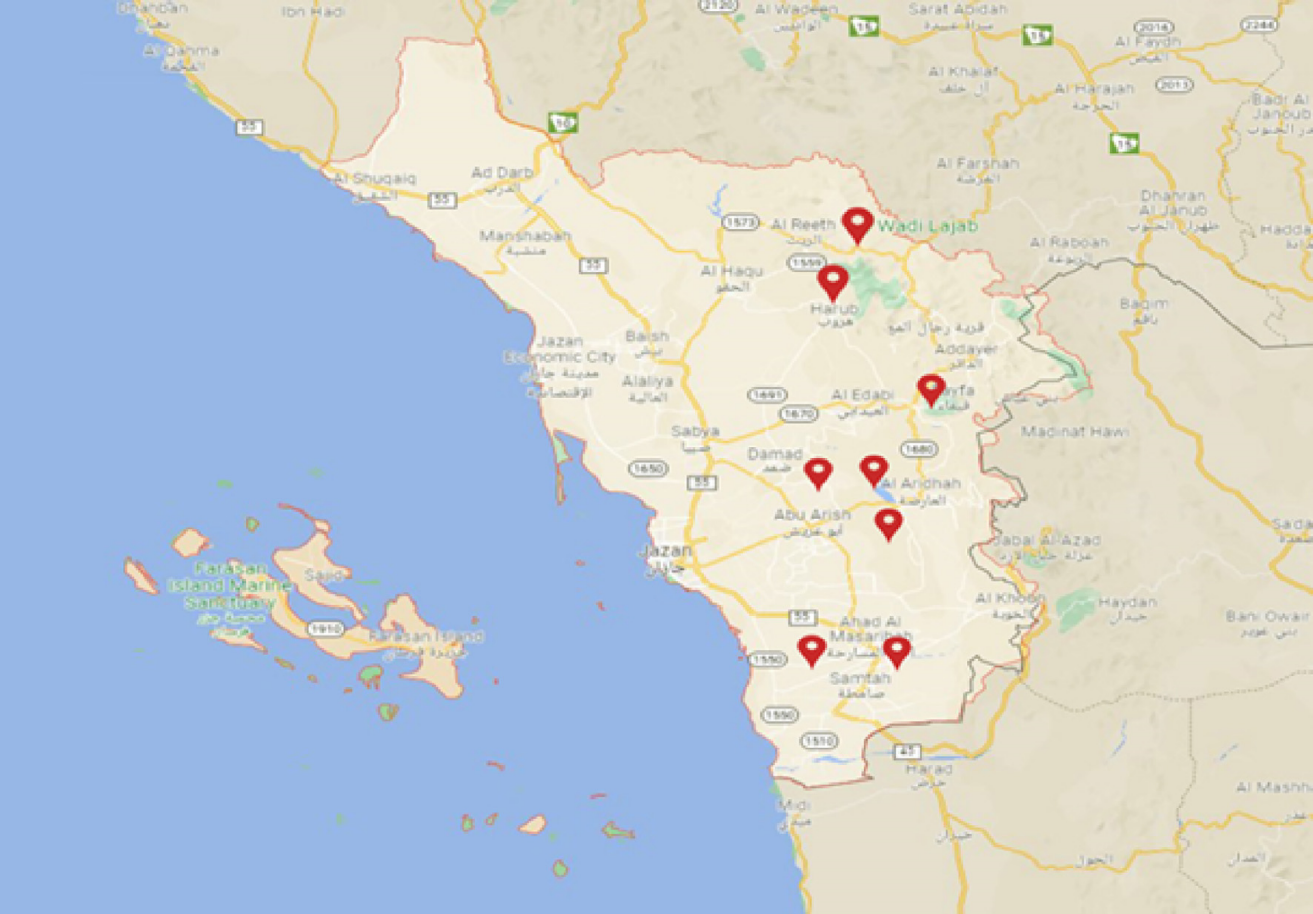
A map of Jazan, Saudi Arabia indicating the collection sites of soil samples.

The geo-referencing of the collection places was done via GPS (Global Positioning System) and chosen from different plants located in eight agricultural areas in Jazan, Saudi Arabia in September 2020 to represent all the different areas (Fig. 1). Twenty-four rhizosphere soil samples were selected for the isolation of bacteria to represent all the different areas according to the soil type and sampling distribution. GPS was used to geo-reference the collecting points. Soil samples (1 0 0) were collected with clean shovels in clean plastic bags. Samples were taken from the rhizosphere area (5 cm below the surface) and then transported to the laboratory within 12 h and analyzed the same day. The temperature during collection was measured by a laboratory thermometer and recorded. Twenty-four rhizosphere soil samples were selected for the isolation of bacteria to represent all the different areas (8 agricultural regions) according to the soil type and sampling distribution. Aseptically, five grams of each selected soil sample was suspended in 100 ml sterile normal saline (0.9%) and homogenized with a magnetic stirrer for 10 min.

### 2.2 Isolation and characterization of bacteria

Suspended soil samples were subjected to aseptic dilution individually by serial dilution method then 100 ml of each mixed diluted solution tube was spread on the surface of prepared T3 medium plates (Travers et al., 1987). Inoculated plates were incubated for 24 h at 30 °C. Bacterial colonies were subjected to steaking on the sterilized T3 medium plates for further purification. According to macroscopic examination; colonial color (white to off-white), texture margin, and size, the separate colonies (100 isolates) were chosen and then transferred aseptically to T3 medium agar plates. After incubation at 30 °C for 24 h, the bacteria were subjected to a gram stain, endospore stain, and biochemical tests to confirm the identification of bacterial isolates. Out of 100 isolates, four isolates selected coded JZ1, JZ2, JZ3, and JZ4. Coded isolates JZ1, JZ2, JZ3, and JZ4 were isolated from Wadi Khalb-Samatah (Khlab), Ayash-Abuarish (Ayash), Abu Arish and Al Saahlil respectively, Jazan Region, Saudi Arabia.

The reference bacterial strain used in the current study, *Bacillus thuringiensis* subsp. *kurstaki* HD1 (BTSK) was obtained from Microbial Molecular Biology Laboratory, Agricultural Genetic Engineering Research Institute, Agricultural Research Center, Egypt.

### 2.3 Staining of endospore and parasporal inclusion bodies

One hundred isolates were subjected to endospore stain, then examined microscopically by phase-contrast microscope to examine the presence of endospores and parasporal inclusion bodies. For the preparation of bacterial suspension began with a separate colony of purified isolate inoculated aseptically in broth (25 ml) T3 medium, then incubated in a shaker incubator (100 rpm) at 30 °C for 72 h. One ml of each incubated broth suspension was centrifuged (4500 rpm) for 10 min. The supernatant was discarded, and the pellet was suspended in 1 ml sterile normal saline. A loopful of suspended bacterial suspension was used to form bacterial film, heat fixing, subjected to spore stain procedures, and then examined by an oil immersion lens of the light microscope.

### 2.4 Molecular characterization

The universal primers 27F and 1492R were used to amplify 16S rRNA PCR (Chen et al., 2012). The designed primers were created using the conserved portions of the 16S rDNA sequences that were readily available. The Lepdopterain *Cry1* gene was targeted by 2 pairs of primers according to Carozzi et al., (1991). The processing of the reaction was acquired by single step thermal cycler starting with Pre-denaturation (at 94° C for two minutes), followed by 35 cycles of denaturation at 94° C for one minute, annealing at 50° C for one minute, extension at 72° C for one minute, and final extension at 72° C for five minutes after that NCBI Blastn was used to sequence and analyze the amplified sequences.

### 2.5 Protein electrophoresis

Analysis of protein samples was performed by SDS-PAGE (sodium dodecyl sulfate–polyacrylamide electrophoresis formulated by gel 4% stacking, 10% separating gel). The Bio-Rad vertical system was used for electrophoresis, and it was charged to 150 V with 1 × run buffer (25 mM Tris-base, 35 mM SDS, and 1.92 mM glycine).

### 2.6 Insecticide larvae bioassay

Isolated *B. thuringiensis* strains were tested on *Aedes aegypti* (2nd larval stage) and *Spodoptera littoralis* (1st larval stage) according to Aranda et al., (1996). Degrades suspension concentrations of spore-tested bacterial suspensions were prepared by serially dilution methods and then formulated with an insect diet. The mortality rate and LC_50_ calculated confidence limits according to probit analysis (Finney, 1981, Soares-da-Silva et al., 2015) after the recorded observation.

### 2.7 Data analysis

Using NCBI, 16S rRNA sequences of the sleeted *B. thuringienssi* isolates were uploaded one by one in query form in comparison to already reported sequences in Genbank using the Basic Local Alignment Search Tool (BLASTn). The sequencing data acquired for 16S rDNA*,* of selected *B. thuringiensis* isolate, were aligned separately using CLUSTALW through MEGA 6.0 (Tamura et al., 2013).

### 2.8 Statistical analysis

Using IPMSPSS version 19.0 and *p* values < 0.05 as a significant level, two-way ANOVA with analysis of variance was performed on the data. According to Snedecor and Cochran (1982), the Duncan test is used to determine whether variations in sample means are significant.

## 3 Results

### 3.1 Isolation and identification of *Bacillus* isolates

One hundred bacterial isolates have been isolated and purified from a total of rhizosphere soil samples. The temperature of the sampling day ranged from 35 − 36 °C. One hundred separated colonies were chosen according to macroscopic examination suspected to be *Bacillus thuringiensis,* then each colony was sub-cultured into T3 medium. After the incubation period, the freshly cultivated purified bacterial cultures were subjected to gram stain, endospore stain, and biochemical tests to confirm the identification of bacterial isolates. Out of 100 isolates, four isolates selected coded JZ1, JZ2, JZ3, and JZ4.

The results of the microscopic examination revealed that isolates JZ1, JZ2, JZ3, and JZ4 are spore-forming bacteria. No major differences between JZ1 and JZ2 cells have been observed; every two isolates contain central endospore and parasporal crystalline inclusion bodies. Whereas, both JZ3 and JZ4 contain only one terminal endospore and were identified biochemically as *B. subtilis* and *Bacillus paramycoides* respectively.

### 3.2 Molecular characterization

Sequencing of the 16S rRNA to characterize the molecular makeup of *BT*.

**Fig. 2:**
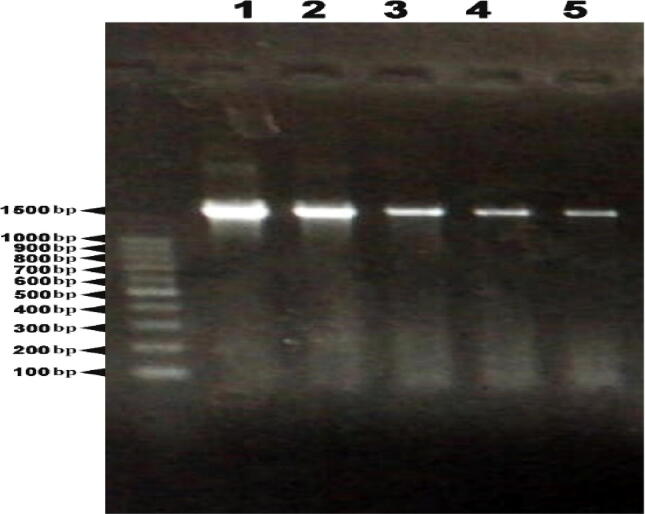
Agarose gel (1%) electrophoresis for 16S rRNA PCR. Lane M: DNA MW Marker, Lane 1: *B. thuringiensis* subsp. *kurstaki*, Lane 2: JZ1 isolate, Lane 3: JZ2 isolate, Lane 4: JZ3 isolate and Lane 5: JZ4 isolate.

The 16S rDNA amplification was carried out using universal primers, the two local isolates (JZ1 and JZ2), and the reference strain of BTSK exhibiting the expected amplicon of 1400 bp (Fig. 2, lanes, 1, 2, and 3). Each isolate's amplified products were sequenced and put through an analysis using NCBI BLAST software. The 16S rDNA sequences of the existing BTSK were found to be completely identical by BLAST results. The two isolates (JZ1 and JZ2) nucleotide sequences were recorded in the gene bank under accession numbers ON799378 and ON799400 respectively.

### 3.3 SDS-PAGE analysis of sporulated and vegetative bacterial forms

**Fig. 3:**
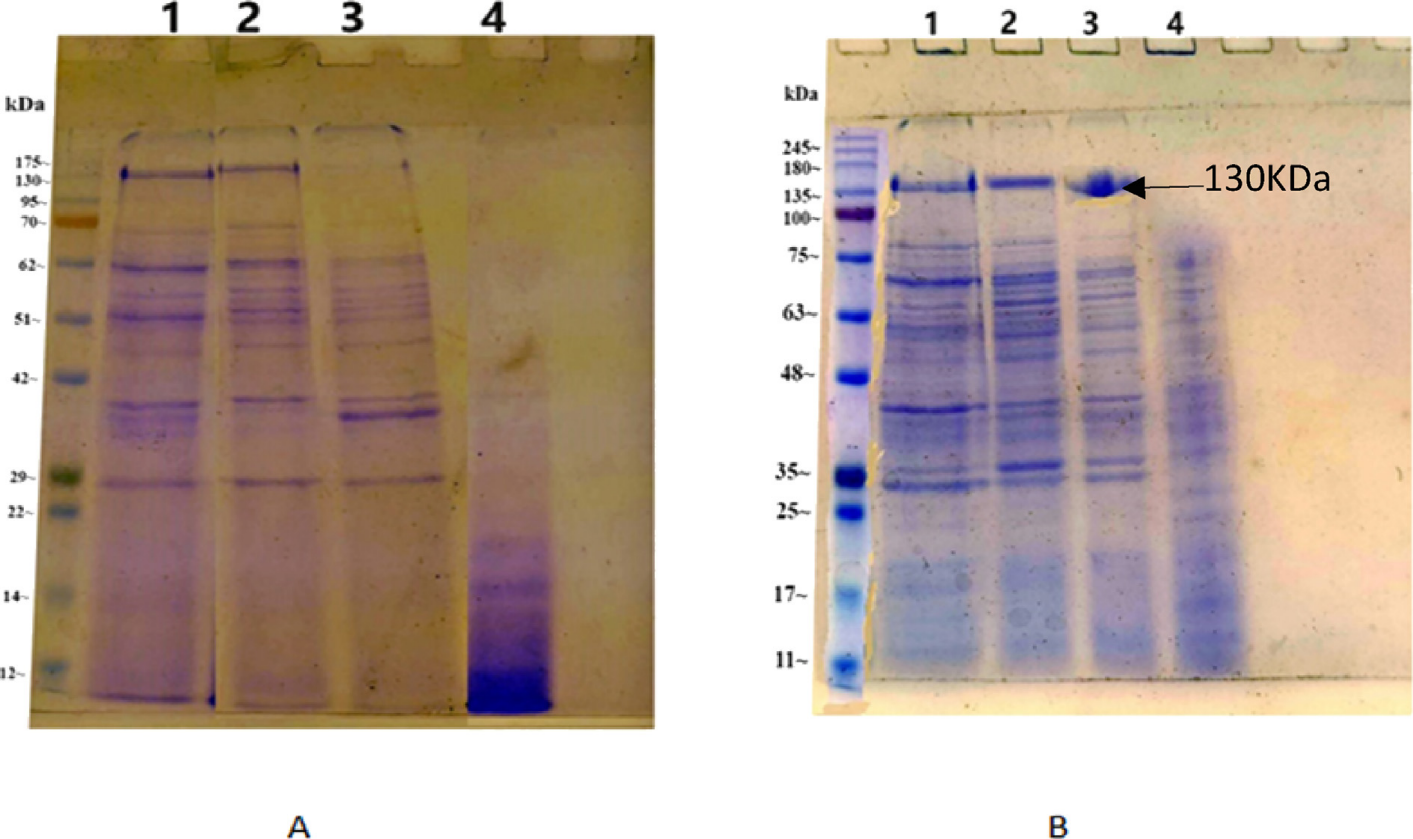
Cellular proteins SDS-PAGE analysis of vegetative (panel A) and sporulated (panel B) cell forms of JZ1 & JZ2 isolates, and standard strain (BTSK), (stained with coomassie brilliant blue). Lane M; high molecular weight protein standard, Lane 1; *B.t kurustaki HD1*, Lane 2; JZ1, Lane 3; JZ2, and Lane 4; JZ3.

The total cellular proteins of the isolates JZ1 and JZ2 vegetative and sporulated cells were subjected to fractionation of denaturing fragments on electrophoresis (SDS-PAGE) and protein bands were visualized by staining with coomassie brilliant blue R-250. The protein banding patterns of the two isolates and reference strain at vegetative growth were compared. The results exhibited that the two isolates were different from each other illustrated in panels A and B (Fig. 3). Moreover, the banding patterns of JZ1, JZ2, and standard authentic BTSK *HD1* were almost identical with some minor differences as shown in panels A and B (Fig. 3).

A proximately 130 KDa protein band was seen for the JZ1 and JZ2 isolates in the sporulated cell form. This common band migrated in a position similar to the toxin bands produced by BTSK HD1, illustrated in panel B lanes 1, 2, and 3 (Fig. 3). The protein profile of JZ3 was completely different from the BTSK, JZ1, and JZ2.

### 3.4 Detection of the *lepidopteran* gene (*cry1*) in JZ1 and JZ2 isolates

**Fig. 4:**
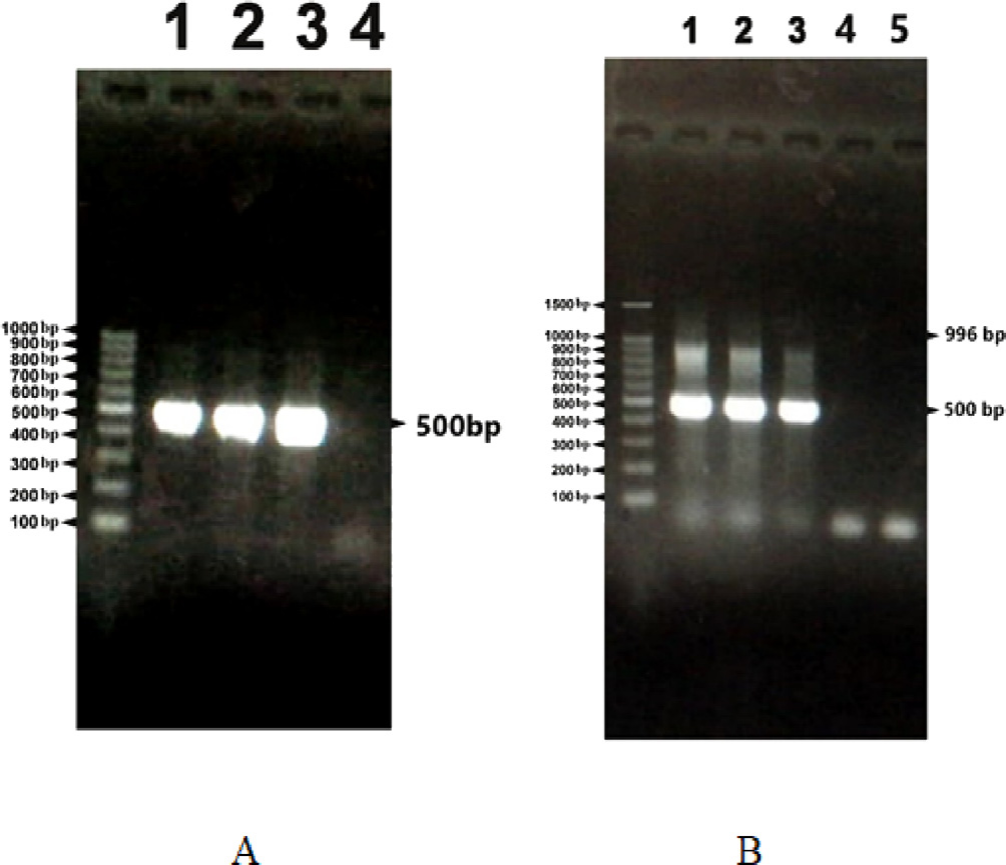
PCR amplification of *Cry1* gene using Lep 1A, Lep 2A, Lep 1B, and Lep 2B primers, the size of the PCR product was 500 bp and 996 bp. Lane M; 100 bp DNA marker. Panel A contains Lane 1; BTSK, Lane 2; JZ2, Lane 3; JZ2 with Lep 1A and Lep 1B. Panel B contains Lane 1; BTSK, Lane 2; JZ2, Lane 3; JZ2 with Lep 2A and Lep 2B.

**Fig. 5:**
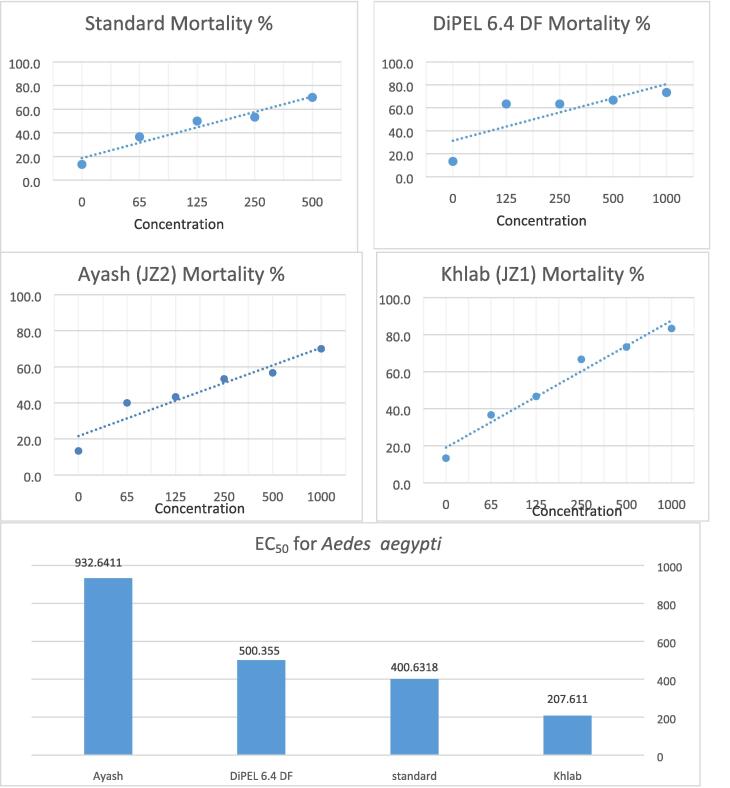
Mortality percentages of *Aedes aegypti* 2nd instar larvae treated with different concentrations of *BT* isolates (Khlab JZ1 and Ayash JZ2), BTSK, and the commercial bio-pesticide DiPEL 6.4 DF.

**Fig. 6:**
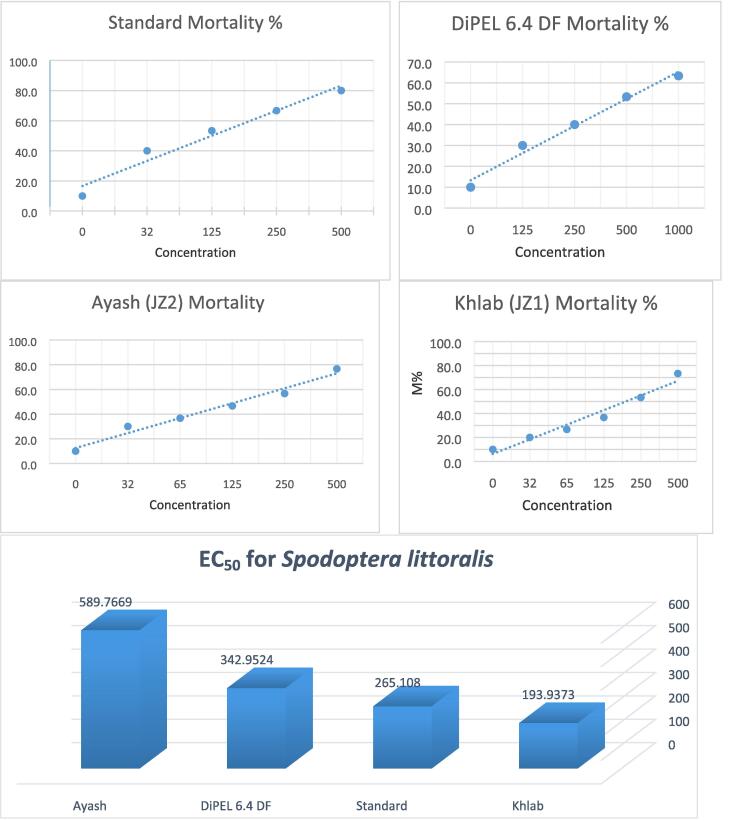
Mortality percentages of *Spodoptera littoralis* 1st instar larvae treated with different concentrations of *BT* isolates (Khlab JZ1 and Ayash JZ2), BTSK, and the commercial biopesticide DiPEL 6.4 DF.

In this study, we used universal primers reported by Hassan et al., (2021) to detect the *Cry1* gene in the local isolates of *B. thurigiensis* JZ1 and JZ2. *lepidopteran*- universal primers LeplA and LeplB yield a 490-bp PCR product with isolates JZ1, JZ2, and reference strain BTSK (Fig. 4), panel A, lanes 1, 2, and 3. While, the mix of the four primers Lep1A, Lep1B, Lep2A, and Lep2B gave the two expected PCR products 490 bp and 986 bp (Fig. 3, Fig. 4, Fig. 5, Fig. 6), panel B Lane 1, 2, and 3.

### 3.5 Bioassay of JZ1, JZ2 isolates, and BTSK against insect larvae

a. Toxicity of JZ1 and JZ2 isolates against 2nd larva of *Aedes aegypti*

The results of the bioassay indicated that; the mortality percentages of BT isolates (JZ1 and JZ2), BTSK*,* and the commercial biopesticide DiPEL 6.4 DF were evaluated for toxicity against 2nd instar larvae of *Aedes aegypti* (Table 1). The total larval mortality at bacterial concentration ranged between 65–––1000 ppm within 6 days, and percentages of mortality ranged between 36.7–––83.3%. The concentrations of 65 and 125 ppm recorded insignificant mortality for the tested BT isolates and commercial DiPEL 6.4 DF. In all treatments, the best results were achieved on the fourth day. The results showed that the EC_50_ of *B. thuringiensis* isolates Khlab JZ1 and Ayash JZ2, BTSK, and the commercial biopesticide DiPEL 6.4 DF were 207, 932, 400, and 500 ppm respectively (Table 1 and Fig. 5). The isolate JZ1 recorded the highest mortality while isolate JZ2 was given the lowest mortality. The affected was significant (p < 0.00) by all treatments and concentrations.

b. Toxicity of BT isolates against 1st larvae of *Spodoptera littoralis*

**Table 1:** Effect of *B. thuringiensis* isolates, BTSK, and DIPEL 6.4 DF on mortality of 2nd larval stage of *Aedes aegypti* and 1st larval stage of *Spodoptera littoralis* under lab conditions.

**Tested material**	**Conc.**	**2nd larval stage of *Aedes aegypti***						**1st larval stage of Spodoptera *littoralis***				
		**Larval mortality after a number of days**				**Total larval mortality**		**Larval mortality after a number of days**			**Total larval mortality**	
		**1**	**2**	**4**	**6**	**No.**	**%**	**1**	**3**	**7**	**No.**	**%**
**Control**	0.0	0.33^a^	0.67^b^	0.33^b^	0.00^b^	1.33^c^	13.33^c^	0.33^b^	0.33^b^	0.33^b^	1.00^c^	10.00^c^
**Standard bacteria**	65	0.00^a^	0.33^b^	1.67^a^	1.67^a^	3.67^b^	36.67^b^	1.00a^b^	1.33a	1.67a	4.00^b^	40.00^b^
	125	0.33^a^	1.00^b^	2.33^a^	1.33^a^	5.00^b^	50.00^b^	1.67^a^	1.67^a^	2.00^a^	5.33^b^	53.33^b^
	250	0.33^a^	0.67^b^	2.33^a^	2.00^a^	5.33^b^	53.33^b^	1.33^a^	2.33^a^	3.00^a^	6.67 ^a^	66.67^a^
	500	0.33a	1.33^a^	2.67^a^	2.67^a^	7.00 ^a^	70.00^a^	1.67^a^	3.00^a^	3.33^a^	8.00 ^a^	80.00^a^
**DiPEL 6.4 DF**	125	0.33^a^	1.67^a^	2.33^a^	2.00^a^	6.33 ^ab^	63.33^ab^	0.33^b^	1.33^a^	1.33^a^	3.00^b^	30.00^b^
	250	0.67^a^	1.67^a^	2.33^a^	1.67^a^	6.33 ^ab^	63.33^ab^	0.67^b^	1.67^a^	1.67^a^	4.00b	40.00b
	500	1.00^a^	2.00^a^	2.33^a^	1.33^a^	6.67 ^a^	66.67^a^	1.67^a^	1.67^a^	2.00^a^	5.33^b^	53.33^b^
	1000	0.67^a^	2.33^a^	2.33^a^	2.00^a^	7.33 ^a^	73.33^a^	1.33^a^	2.00^a^	3.00^a^	6.33 ^a^	63.33^a^
**Ayash (**JZ2**)**	65	0.00^a^	1.00^b^	1.67^a^	1.33^a^	4.00^b^	40.00^b^	0.33^b^	1.33^a^	1.33^a^	3.00^b^	30.00^b^
	125	0.33^a^	1.67^a^	1.67^a^	0.67^ab^	4.33^b^	43.33^b^	1.00^ab^	1.33^a^	1.33^a^	3.67^b^	36.67^b^
	250	0.67^a^	2.00^a^	2.00^a^	0.67^ab^	5.33^b^	53.33^b^	0.33^b^	2.00^a^	2.33^a^	4.67^b^	46.67^b^
	500	0.33^a^	2.33^a^	2.33^a^	0.67^ab^	5.67^b^	56.67^b^	0.67b	2.33^a^	2.67^a^	5.67 ^ab^	56.67^ab^
	1000	0.00^a^	2.00^a^	2.33^a^	2.67^ab^	7.00 ^a^	70.00^a^	1.00^ab^	3.00^a^	3.67^a^	7.67 ^a^	76.67^a^
**Khlab (**JZ1**)**	65	0.33^a^	1.67^a^	1.67^a^	0.00^b^	3.67^b^	36.67^b^	0.33^b^	0.33^b^	1.33^a^	2.00 ^bc^	20.00^bc^
	125	0.33^a^	1.67^a^	1.33^a^	1.33^a^	4.67^b^	46.67^b^	0.33^b^	1.33^a^	1.00^ab^	2.67 ^bc^	26.67^bc^
	250	0.33^a^	2.00^a^	2.67^a^	1.67^a^	6.67 ^a^	66.67^a^	0.33^b^	1.33^a^	2.00^a^	3.67^b^	36.67^b^
	500	0.67^a^	2.33^a^	2.33^a^	2.00^a^	7.33 ^a^	73.33^a^	0.67^b^	1.67^a^	3.00^a^	5.33^b^	53.33^b^
	1000	0.33^a^	1.67^a^	3.33^a^	3.00^a^	8.33 ^a^	83.33^a^	1.33a	2.67^a^	3.33^a^	7.33 ^a^	73.33^a^
F (*p-value*)												
Treatments		1.07(0.37)	10.2(0.00)	0.71(0.54)	7.8(0.00)	7.8(0.00)	7.8(0.00)	4.9(0.01)	6.6(0.01)	14.4(0.00)	25.2(0.00)	25.2(0.00)
Concentration		0.32(0.90)	2.5(0.04)	5.3(0.001)	6.6(0.00)	27.8(0.00)	27.8(0.00)	3.6 (0.01	13.6(0.00)	31.1(0.00)	59.1(0.00)	59.1(0.00)

The potency of *B. thuringiensis* isolates (JZ1 and JZ2), BTSK, and the commercial biopesticide DiPE 6.4 DF were determined against *Spodoptera littoralis*. The Mortality levels of larvae caused by the different isolates varied from 63.8 to 80%. The results of the bioassay revealed that; the EC_50_ of *B. thuringiensis* isolates Khlab JZ1 and Ayash JZ2, BTSK, and the commercial biopesticide DiPEL 6.4 DF were recorded 193, 589.76, 265, and 342.9 ppm respectively (Table 1 & Fig. 6). Isolate JZ1 was recorded as the highest mortality and isolate JZ2 was given the lowest mortality. The present findings were the first to report that Khlab isolate is a very potent microbial control against 1st larvae of *Spodoptera littoralis*. Whereas this affected was significant (*p* < 0.00) by most treatments and concentrations. According to *p* values of statistical analysis, *Spodoptera litto*ralis have a higher response than *Aedes aegypti* in most recorded days and total larval mortality to bio treatments.

## 4 Discussion

Biopesticides are substances used to control pests and pathogens that are derived from living organisms or natural products. Since they come from natural sources, they stand out as an environmentally friendly tool because they decompose quickly and reduce pollution issues brought on by synthetic pesticides. These products pose tough problems that influence the bioactivities of the active ingredients (Hernandez-Tenorio et al., 2022). It may be helpful to screen samples from various environments to find BT strains with wider host ranges and new toxic properties against pests (Konecka et al., 2007, Baranek et al., 2023). Soil is the principal natural reservoir of BT spores and is currently the preferred source for the isolation of *Bacillus* sp. (Silva et al., 2012, Soares-da-Silva et al., 2015, El-Kersh et al., 2016, Aynalem et al., 2021). The goal of the current study was to identify native BT isolates from the rhizosphere soil of various commercial plants distributed across eight different agricultural regions in Jazan, Saudi Arabia. The local isolates will be used for the development of biocontrol agents to help control mosquito-borne diseases and insect pests. A total of 100 bacterial isolates were isolated from 8 soil samples. Out of the one hundred isolates we selected four bacterial isolates named JZ1, JZ2, JZ3, and JZ4 which exhibit characteristic features of *Bacillus* sp. for further study. To sum up, we found *B. thuringiensis* only in two sites Ayash-Abuarish and Wadi Khalb-Samatah. The presence of *B. thuringiensis* only in two sites in Jazan, Saudi, may be related to, the nature of the soil in this region and the weather. Out of these four isolates, two isolates JZ1 and JZ2 contain parasporal inclusion bodies. Numerous studies rely solely on cristal inclusions to distinguish BT from other *Bacillus* spp. like *B. cereus*, despite criticism of the use of a single character for classification, such as crystal inclusion bodies (Rasko et al., 2005, Bağcıoğlu et al., 2019). Analysis of the 16S rRNA gene's nucleotide sequences is frequently used for taxonomic localization and genus/species identification of bacteria. To positively identify the two bacterial isolates (JZ1 and JZ2), using 16S rRNA. According to BLAST results showed that; the 16S rRNA sequences of the existing *Bacillus thuringiensis* (BT) were completely similar (Pereira et al., 2020). Moreover, these two isolates (JZ1 and JZ2) related to BTSK (*Bacillus thuringiensis* subsp. *Kurstaki*) according to protein cell profiles were measured by SDS-PAGE of sporulated and vegetative form.

In the present study, the BT pro-toxins size range 130 to 140 kDa was observed in both isolates JZ1 and JZ2 in comparison to BTSK*,* these toxins are active against lepidopteran insects. In addition, the presence of a 66 kDa *Cry2* protein in our isolates is active against Lepidoptera and Diptera insects (Höfte and Whiteley, 1989, Abbas, 2018). For further identification and characterization of isolates, universal primers for *Cry1* gene were used to detect the gene *Cry1* in our isolates. In the current study agrees with information from previous studies (Bravo et al., 2007, Hassan et al., 2021). Bioassay remains an essential tool to determine insecticidal activity for instance *Aedes aegypti* and *Spodoptera littoralis* larvae and in the previous studies, these isolated bacteria showed different effects on different types of insects therefore it is used commercially (Şahin et al., 2018, Wu et al., 2021, Ma et al., 2023, Pitton et al., 2023). The insecticidal activity of the isolates JZ1 and JZ2 in comparison to BTSK and commercial products of BT*,* DiPEL 6.4 DF have been studied. We found that the EC_50_ of *B. thuringiensis* isolates Khlab JZ1, Ayash JZ2, BTSK, and the commercial biopesticide DiPEL 6.4 DF against the 2nd instar larvae of *Aedes aegypti* were 207, 932, 400, and 500 ppm respectively. The JZ1 isolate recorded the highest mortality and the local isolate JZ2 was given the lowest mortality. The insecticidal activity of our isolates against *Aedes aegypti* is due to the presence of *Cry2* toxin in these isolates which is active against Lepidopteran and Dipteran insects. The EC_50_ of *B. thuringiensis* isolates JZ1, JZ2, BTSK, and the commercial biopesticide DiPEL 6.4 DF against the first instar larvae of *Spodoptera littoralis* were recorded 193.93, 589.7, 265.108 and 342.9, ppm respectively. Isolate JZ1 recorded the highest mortality and isolate JZ2 was given the lowest mortality. In the context of local development, production, and application of biological control of some lepidopteran (*Spodoptera littoralis*) and dipteran insects (*Aedes aegypti*) insects in Saudi Arabia, a bioassay is promising.

Based on the results shown above, the study reached important results in isolating and characterization of *Bacillus thuringiensis* one of the elements of biological pest control. Isolated organisms are considered an important addition, as they are endemic and adapted to the special environment of Jazan, where the humidity and temperature are high. Therefore, the study recommends completing experiments in using these organisms on economic pests in the Jazan region if the need arises to combat them.

## 5 Conclusion

*Bacillus thuringiensis* affects specific pests and does not harm beneficial insects or other organisms. Therefore, pesticides depending on *Bacillus thuringiensis* are regarded as environmentally friendly in comparison to synthetic pesticides, they are also less hazardous to both humans and animals. This work produced significant findings about the isolation and characterization of *Bacillus thuringiensis.* Since isolated organisms are indigenous and have evolved to thrive in the unique high humidity and temperature habitat, they are seen as a valuable addition. Therefore, it is necessary to test the effect of isolates on additional pests because it suggests concluding experiments utilizing these organisms on economic pests in the Jazan region and other similar environmental areas in Saudia Arabia.

## Declaration of Competing Interest

The authors declare that they have no known competing financial interests or personal relationships that could have appeared to influence the work reported in this paper.
